# Splicing-Dependent RNA Polymerase Pausing in Yeast

**DOI:** 10.1016/j.molcel.2010.11.005

**Published:** 2010-11-24

**Authors:** Ross D. Alexander, Steven A. Innocente, J. David Barrass, Jean D. Beggs

**Affiliations:** 1 Wellcome Trust Centre for Cell Biology, University of Edinburgh, King's Buildings, Edinburgh EH9 3JR, UK; 2 Edinburgh Centre for Systems Biology, University of Edinburgh, King's Buildings, Edinburgh EH9 3JR, UK

## Abstract

In eukaryotic cells, there is evidence for functional coupling between transcription and processing of pre-mRNAs. To better understand this coupling, we performed a high-resolution kinetic analysis of transcription and splicing in budding yeast. This revealed that shortly after induction of transcription, RNA polymerase accumulates transiently around the 3′ end of the intron on two reporter genes. This apparent transcriptional pause coincides with splicing factor recruitment and with the first detection of spliced mRNA and is repeated periodically thereafter. Pausing requires productive splicing, as it is lost upon mutation of the intron and restored by suppressing the splicing defect. The carboxy-terminal domain of the paused polymerase large subunit is hyperphosphorylated on serine 5, and phosphorylation of serine 2 is first detected here. Phosphorylated polymerase also accumulates around the 3′ splice sites of constitutively expressed, endogenous yeast genes. We propose that transcriptional pausing is imposed by a checkpoint associated with cotranscriptional splicing.

## Graphical abstract

**Graphical Abstract:**

**Figure:**
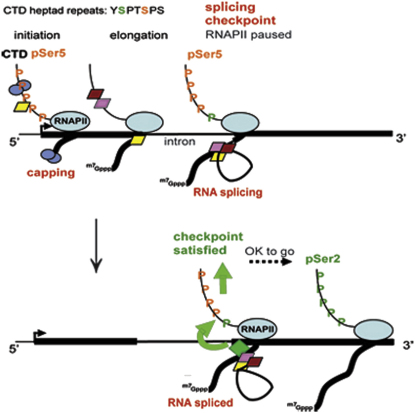

**Highlights:**

► RNA polymerase II pauses at the 3′ end of an intron ► RNA polymerase II pausing depends on productive splicing ► Dynamic phosphorylation of polymerase changes according to intron splicing status ► We propose a transcriptional checkpoint that responds to cotranscriptional splicing

## Introduction

Most transcripts produced by eukaryotic RNA polymerase II (RNAPII) undergo processing at their 5′ ends (capping), 3′ ends (cleavage and polyadenylation), and internally (splicing to remove introns). It is now widely accepted that many of these modifications, especially capping and 3′ end formation, occur cotranscriptionally, that is, before transcription is completed and the RNA is released from the site of transcription (Pandit et al., 2008; Perales and Bentley 2009). Splicing can occur co- or posttranscriptionally. For example, early electron micrographs of *Drosophila* cells showed that many but not all introns are removed cotranscriptionally (Beyer and Osheim, 1988). The chromatin immunoprecipitation (ChIP) method has been used to demonstrate the sequential recruitment of spliceosomal small nuclear ribonucleoprotein particles (snRNPs) to the site of transcription, indicating spliceosome assembly on nascent transcripts in both yeast and mammalian cells (Görnemann et al., 2005; Lacadie and Rosbash, 2005; Listerman et al., 2006; Tardiff et al., 2006; Moore et al., 2006). The carboxy-terminal domain (CTD) of RNAPII large subunit binds a number of RNA processing factors, including capping enzymes, certain splicing factors, and 3′ end processing factors (Buratowski, 2009). Thus, one explanation for the occurrence of cotranscriptional RNA processing could be that it is simply a consequence of the cotranscriptional recruitment of the factors involved. However, there is evidence, at least in mammalian systems, that coupling between the different processes is functionally important (Kornblihtt et al., 2004; Neugebauer, 2006; Pandit et al., 2008; Perales and Bentley, 2009).

The CTD consists of multiple repeats (52 in mammals and 26 in budding yeast) of the heptapeptide sequence YSPTSPS that is dynamically phosphorylated during cycles of transcription (Buratowski, 2009). Typically, the CTD is in a hypophosphorylated form at the promoter. At initiation of transcription, the serine at position 5 (Ser5) becomes phosphorylated to permit promoter clearance. This also promotes recruitment of the capping enzymes. As RNAPII elongates toward the 3′ end of the gene, Ser5 phosphorylation declines and there is increased phosphorylation of Ser2, which promotes recruitment of the 3′ end processing factors (Licatalosi et al., 2002). Recently, Ser7 in the CTD was found to be phosphorylated in a manner resembling that of Ser5 (Egloff et al., 2007; Kim et al., 2009), in addition to which, promoter-distal phosphorylated Ser7 (pSer7) was found on genes transcribed by RNAPII (Tietjen et al., 2010). Currently, the only characterized function for pSer7 is related to 3′ end processing of small nuclear RNAs (snRNAs) (Egloff et al., 2007). Therefore, the CTD was proposed to function as a “landing pad” for the recruitment of RNA processing factors, with the specificity being determined by a “CTD code” of posttranslational modifications (Egloff and Murphy, 2008). Furthermore, the dynamic phosphorylation state of RNAPII is thought to play an important role in the regulation of splicing (Batsché et al., 2006; Kornblihtt, 2006; Pandit et al., 2008).

Changing the dynamics of transcript elongation can also influence downstream processing events and can affect constitutive or alternative splicing (Kadener et al., 2001; Nogues et al., 2002; de la Mata et al., 2003; Howe et al., 2003). It was proposed that the rate of transcriptional elongation can affect inclusion or skipping of an alternative exon by controlling the duration of a “window of opportunity” during which the splicing machinery can recognize an upstream splice site and assemble a spliceosome before the appearance of a competing downstream splice site (Kornblihtt et al., 2004; Perales and Bentley, 2009). Conversely, splicing signals and splicing factors can enhance transcriptional activity (Furger et al., 2002; Damgaard et al., 2008; Lin et al., 2008), suggesting reciprocal interactions.

Here, we present high-resolution kinetic analyses of transcription and splicing, using a series of reporter genes in budding yeast. We demonstrate repeated splicing-dependent transcriptional pausing at the 3′ end of the introns in two different reporter genes. The CTD of the paused polymerase is hyperphosphorylated on Ser5 compared to its status at the promoter, and it becomes phosphorylated on Ser2 at this point. We also demonstrate elevated levels of phosphorylated RNAPII over the 3′ splice sites of several endogenous yeast genes, suggesting that this is a general phenomenon. We propose that transcriptional pausing is imposed by a checkpoint that is associated with cotranscriptional splicing. We discuss candidate proteins that might function as checkpoint factors and speculate that multiple checkpoints may exist that correspond to surveillance mechanisms operating at different stages of the splicing process.

## Results

**Figure 1:**
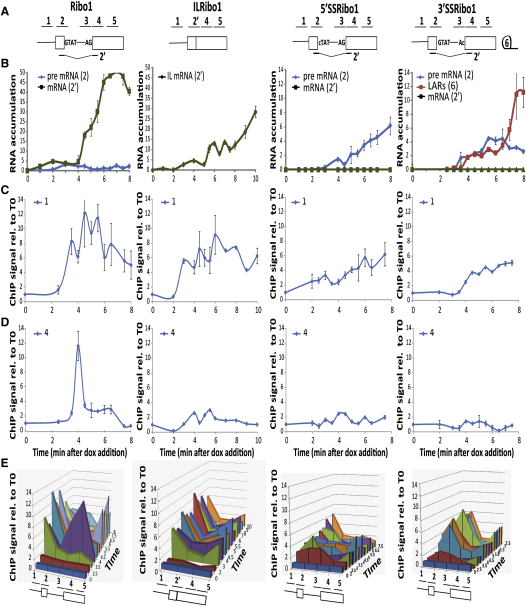
Analysis of Ribo Reporter Induction (A) Diagram of Ribo1, ILRibo1, 5′SSRibo1, and 3′SSRibo1 genes. Exons are represented by rectangles, and the intron by a line with sequences at the ends. The reporter genes are based on previously described *ACT1-PGK1* constructs (Hilleren and Parker, 2003; Alexander et al., 2010) expressed under control of a *tetO7-CYC1-UAS* promoter (Bellí et al., 1998). The lines above indicate amplicons analyzed in RT-qPCR or ChIP analyses: 1, 2, 3, 4, and 5 correspond to the promoter, exon 1/5′SS, 3′SS, 5′ end of exon 2 and 3′ end of exon 2, respectively. Amplicon 2′ corresponds to spliced Ribo1 mRNA or the 5′ end of the intronless ILRibo1. Lariat introns (6) were assayed with an oligo that hybridizes across the 2′-5′phosphodiester bond at the branch site (Vogel et al., 1997). See the Supplemental Information for full details of strains, reporter genes, and primer sequences. (B) RT-qPCR analysis of accumulation of pre-mRNA, mRNA, or lariat RNA species (amplicon indicated in parentheses) showing the increase compared to time of doxycyclin addition (T0). Error bars indicate standard error for RT performed in triplicate and qPCR also performed in triplicate. (C) ChIP analysis (presented as percentage of input relative to uninduced level at T0) to detect RNAPII at the promoter (amplicon 1) using anti-Rpb3 antibodies (Neoclone) with the same cultures as above. (D) ChIP analysis to detect RNAPII at the 5′ end of exon 2 (amplicon 4), otherwise as above. (E) 3D representation of the RNAPII ChIP data, showing RNAPII occupancy at all positions tested (x axis) at times indicated on the z axis. In ChIP assays, error bars indicate standard error for qPCR performed in triplicate. The kinetic resolution of the ChIP assay was tested by RT-qPCR measurement of Ribo1 transcription during the formaldehyde treatment, showing that transcript accumulation ceases immediately, and indicating that RNAPII activity is halted very rapidly (data not shown). See also Figure S1.

To follow the in vivo kinetics of pre-mRNA splicing in *Saccharomyces cerevisiae*, we integrated reporters based on hybrid *ACT1-PGK1* sequences (Hilleren and Parker 2003; Alexander et al., 2010) into the genome at the *HIS3* locus under either tetracycline-inducible (tetON) or tetracycline-repressible (tetOFF) control (Bellí et al., 1998). Both the tetON and tetOFF strains express tetracycline-responsive repressor and tetracycline-responsive transactivator proteins, which provides a good dynamic range of gene expression (Bellí et al. [1998]; in this work, the tetracycline analog doxycyclin was used). The 1.3 kb Ribo1 gene (Figure 1A; see the Supplemental Experimental Procedures available online for full details) contains the budding yeast *ACT1* intron with a short insertion to allow the transcripts to be distinguished from endogenous *ACT1* transcripts in reverse transcriptase quantitative real-time PCR (RT-qPCR) assays. Variants of this reporter contain a point mutation at the 5′ splice site (5′SSRibo1), 3′ splice site (3′SSRibo1), or branch site (BSRibo1) or lack an intron (ILRibo1) (Hilleren and Parker 2003; Alexander et al., 2010). Addition of doxycyclin to the growth medium of a tetON Ribo1 strain resulted in the transient low level accumulation of Ribo1 pre-mRNA at about 3 min, followed by spliced Ribo1 mRNA from 4 min, indicating splicing activity (Figure 1B, Ribo1). Similarly, ILRibo1 mRNA was detectable from 3 min (Figure 1B, ILRibo1).

ChIP assays, using antibodies against the Rpb3p subunit of RNAPII, detected RNAPII recruitment to the promoter region of the Ribo1 and ILRibo1 genes by 3 min after doxycyclin addition, and the level of RNAPII at the promoter then remained above the uninduced level (Figure 1C; qPCR amplicon 1, indicated in Figure 1A). At 4 min (when spliced mRNA was first detectable), a strong, transient accumulation of RNAPII was observed around the 3′SS and just downstream at the 5′ end of exon 2 of Ribo1 (amplicons 3 and 4; Figures 1D and 1E; see Figure S1 for the full dataset) but not in the corresponding region of ILRibo1 (amplicon 4; Figures 1D and 1E). Note that the Rpb3p ChIP signal is low over the exon1/5′SS region (amplicon 2), showing that RNAPII levels on either side, at the promoter and 3′SS, can be distinguished in this assay (Ribo1 in Figure 1E and Figures S1A–S1C). From 5.5 min, there was also a persistent RNAPII signal toward the 3′ end of Ribo1 that was consistently higher than the signal at the 3′ end of ILRibo1, although the latter appeared earlier, from 3 min (amplicon 5; Figure 1E). These results were highly reproducible and were similar upon derepression of tetOFF strains (data not shown). Although the timing of the first detection of transcripts varied slightly between different cultures, the transient accumulation of RNAPII in the 3′SS region always coincided with the appearance of spliced Ribo1 mRNA. In several experiments, two peaks of RNAPII were detected at or near the 3′ splice site, a few minutes apart (e.g., Figure S1C).

We next tested the effect of point mutations at the 5′SS or 3′SS (Figure 1A), which abolish the first or second step of splicing, respectively. Unspliced 5′SSRibo1 transcripts accumulated from about 3 min and, as expected, no spliced mRNA was detectable (Figure 1B, 5′SSRibo1). 3′SSRibo1 RNA is a substrate for the first but not the second step of splicing, and the lariat intron-exon 2 product of the first step accumulated with a delay of about 30 s after the appearance of pre-mRNA (Figure 1B, 3′SSRibo1). ChIP assays showed RNAPII accumulation at the promoter of each mutant reporter gene (Figure 1C), but to a lower level than with the Ribo1 gene, suggesting reduced transcriptional activity, and there was no accumulation of RNAPII around the 3′SS (Figures 1D and 1E; amplicons 3 and 4). Thus, the transient RNAPII accumulation around the 3′SS region of the Ribo1 gene depends on the presence of a fully functional intron and/or completion of the splicing reaction, and neither spliceosome assembly nor the first step of splicing is sufficient to cause this. However, with the 3′SSRibo1 reporter, we observed a persistent accumulation of RNAPII over the exon1/5′SS (amplicon 2), suggesting that in the absence of a functional 3′SS, there is a change in the dynamics of transcript elongation, with RNAPII slowing its elongation rate or pausing over exon1/5′SS.

**Figure 2:**
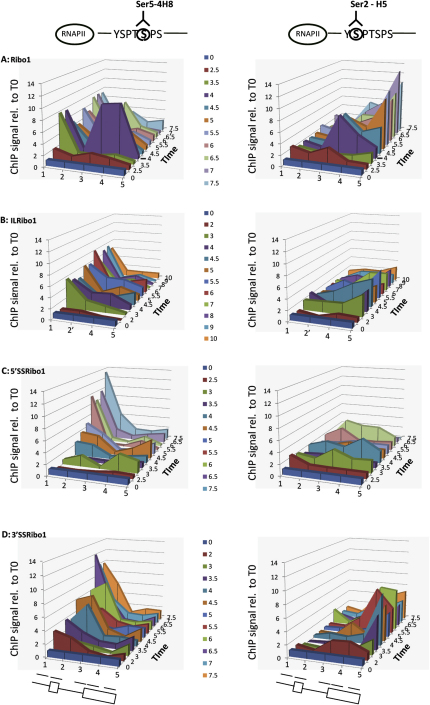
Phosphorylation Status of RNAPII CTD during Induction Top: Diagrams showing RNAPII CTD heptad repeat with antibodies to detect pSer5 (4H8; Millipore) or pSer2 (H5; Covance). (A–D) ChIP analysis to detect RNAPII with pSer5 (left) or pSer2 (right) at different times after dox addition (z axis), at all positions tested (x axis) for Ribo1, ILRibo1, 5′SSRibo1, and 3′SSRibo1 as indicated. Data are plotted in 3D and presented as percentage input relative to T0. Other details are as in Figure 1. See also Figure S2.

The phosphorylation status of the CTD was also monitored by ChIP, using antibodies specific for phosphorylated serine 5 (pSer5) or phosphorylated serine 2 (pSer2) (Kim et al., 2009). This showed that, as expected, RNAPII at the promoter of Ribo1 had mainly pSer5 (Figure 2A). The RNAPII that accumulated transiently around the 3′SS at 4 min was also highly phosphorylated on Ser5, and pSer5 RNAPII accumulated transiently at the 3′SS again a few minutes later (Figure S2C). Notably, there was little or no pSer5 detected between the promoter and the 3′SS (amplicon 2 in Figure 2A, left panel). The paused RNAPII was also phosphorylated on Ser2 (Figure 2A, right panel; Figure S2C), with the 3′SS being the most 5′ position on the gene at which pSer2 was detected. This suggests that phosphorylation of Ser2 occurred on the paused RNAPII; however, the pSer2 accumulation may not display exactly the same timing as the pSer5 data. Toward the 3′ end of the gene, pSer5 declined, whereas pSer2 increased at later time points. Presenting the RNAPII phosphorylation signal as a proportion of the total RNAPII signal shows that RNAPII at the 3′SS was hyperphosphorylated compared to RNAPII at the promoter (Figure S2, pSer5/RNAPII, compare A and C). This suggests new phosphorylation of Ser5 and Ser2 at the 3′SS.

With the intronless ILRibo1 reporter, the pSer5 signal simply decreased from the promoter toward the 3′ end of the gene, as the pSer2 signal gradually increased (Figure 2B). With 5′SSRibo1, RNAPII with pSer5 accumulated strongly at the promoter, despite the lower level of total RNAPII signal, and there was only a low level of pSer2 across the body of the gene, more like ILRibo1 than Ribo1 (Figure 2). With 3′SSRibo1 (Figure 2D), there was an accumulation of pSer5 at the promoter and also over the exon1/5′SS (amplicon 2), compatible with a slowing or pausing of RNAPII in this region. The level of pSer2 increased toward the 3′ end of the 3′SSRibo1 gene. Thus, the dynamics of RNAPII phosphorylation differ significantly with the splicing status of the gene.

If the RNAPII pause in the region of the 3′SS is determined by splicing, two predictions can be made: (1) splicing of Ribo1 transcripts should be cotranscriptional at this time and (2) suppression of the splicing defect of a mutant intron will lead to RNAPII pausing on the mutant gene.

**Figure 3:**
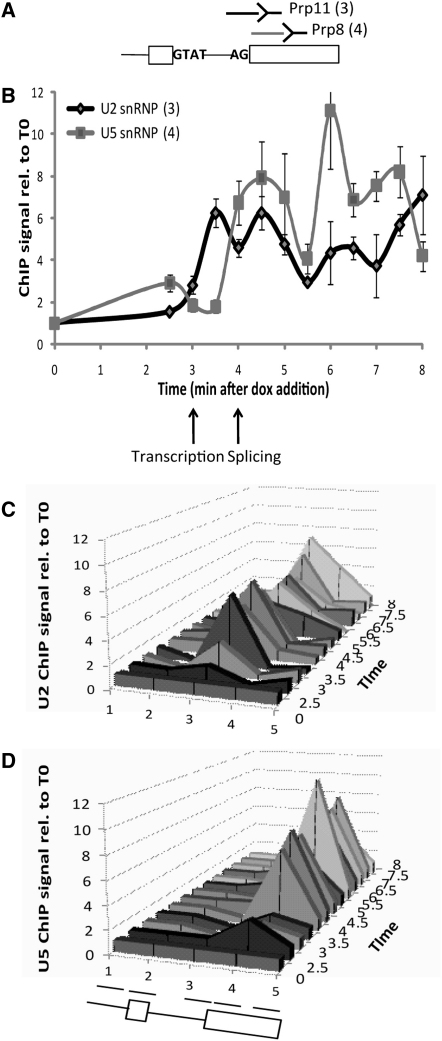
Cotranscriptional Recruitment of Splicing Factors (A and B) The positions on Ribo1 (A) at which recruitment of Prp11p (U2 snRNP; black) or Prp8p (U5 snRNP; gray) was detected by ChIP as shown in (B), using the same culture as in Figure 1B. Error bars indicate standard error for qPCR performed in triplicate. Arrows below indicate the times at which pre-mRNA and spliced mRNA were first detected. (C and D) 3D representations of the ChIP data for Prp11p and Prp8p respectively at different positions on the Ribo1 gene (x axis) and at different times after dox addition (z axis).

To address the first point, we analyzed the cotranscriptional recruitment of U2 and U5 snRNPs by performing ChIP with antibodies to the snRNP components Prp11p and Prp8p, respectively. U2 snRNP was detectable at 3.5 min after doxycyclin addition, and the U5 snRNP was first detected at 4 min (Figure 3), consistent with cotranscriptional spliceosome assembly at the time of the RNAPII pause (Görnemann et al., 2005) and continuing thereafter. Furthermore, we have recently shown in a kinetic analysis of splicing and 3′ end formation that a significant amount of splicing of Ribo1 transcripts occurs prior to 3′ end cleavage and polyadenylation, indicating cotranscriptional splicing (Alexander et al., 2010). It is therefore conceivable that cotranscriptionally recruited splicing factors might affect RNAPII and/or chromatin factors that are in close proximity.

**Figure 4:**
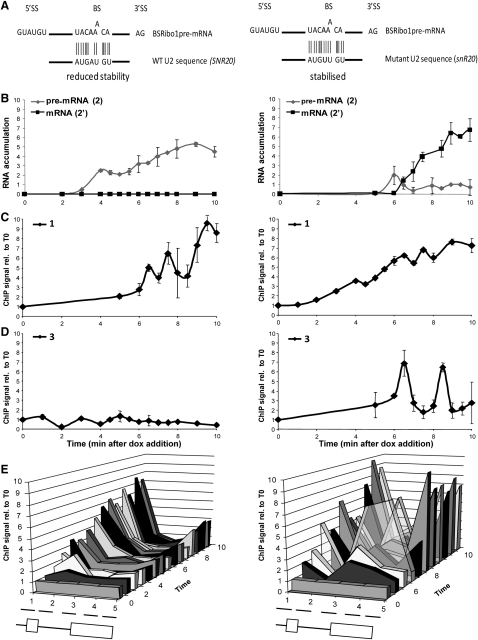
It Is the Splicing Event Rather than the Intron Sequence that Causes RNAPII to Pause at the 3′SS (A) Diagram showing BSRibo1 mutant branchsite sequence base paired to WT U2 snRNA (left) and to mutant U2 snRNA (right). (B) RT-qPCR analysis of BSRibo1 pre-mRNA and spliced mRNA accumulation in the presence of WT (left) and mutant (right) U2. (C–E) ChIP analysis to detect RNAPII on the BSRibo1 gene using anti-Rpb3p antibodies. Details are as in Figures 1C–1E. Error bars indicate standard error for qPCR performed in triplicate. See also Figure S3 for more detail and for ChIP analysis of pSer5 and pSer2.

To test the second prediction, we used the BSRibo1 reporter that has a point mutation at the branch site, which causes a first step splicing defect. This splicing defect can be largely suppressed by a mutant U2 snRNA that restores base pairing with the mutant branch site sequence (Parker et al., 1987) (Figure 4A). Plasmids encoding the wild-type or mutant U2 snRNA were introduced into a tetOFF BSRibo1 strain. After derepression in the wild-type U2 control strain, unspliced transcripts accumulated with no detectable splicing (Figure 4B, left panel), and there was no RNAPII accumulation around the 3′SS (Figures 4D and 4E; amplicons 3 and 4). This resembles the ILRibo1 and 5′SSRibo1 result (Figure 1D). In a strain producing mutant U2 snRNA that complements the BSRibo1 mutation, splicing was substantially restored (Figure 4B, right panel). Importantly, RNAPII accumulated transiently at the 3′SS at 6.5 min, the time when spliced mRNA was first detected, and a second peak of RNAPII appeared at the 3′SS at 8.5 min (Figure 4D right panel; see Figure S3 for more detail and ChIP data for pSer5 and pSer2). Thus, it is clearly the actual process of splicing, rather than the intron sequence, that causes transient RNAPII pausing. Additionally, it may be significant that suppression of the BS splicing defect also resulted in higher levels of pSer2 RNAPII toward the 3′ end of exon 2 (amplicon 5; Figure 4E and Figure S3E) as was noted earlier for Ribo1, suggesting that this too may be splicing dependent.

**Figure 5:**
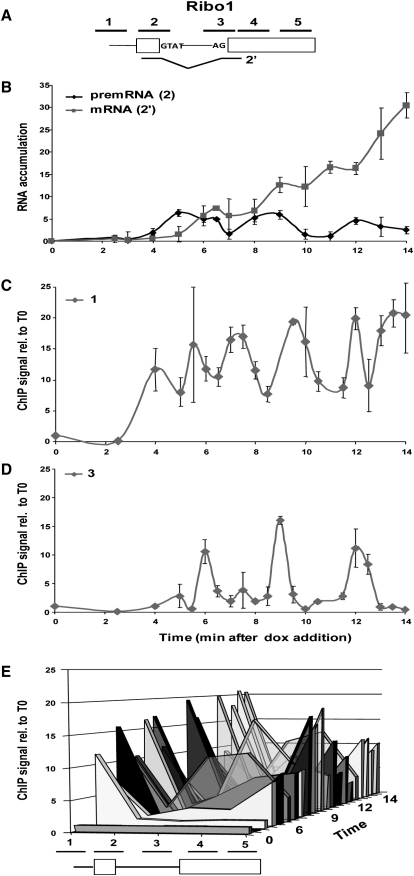
RNAPII Pauses Repeatedly at the 3′ End of the Ribo1 Intron Transcription, splicing, and RNAPII recruitment were analyzed after Ribo1 induction as described in Figure 1 but for a longer period of time. Error bars indicate standard error for qPCR performed in triplicate. For more detail, see also Figure S4.

The observation of a second RNAPII pause in several experiments raised the possibility that pausing may be a recurring event. To test this, we performed a longer time course of Ribo1 induction, and, indeed, RNAPII was observed to accumulate strongly near the 3′SS three times, at approximately 3 min intervals (Figure 5 and Figure S4). Intriguingly, each RNAPII pause seems to occur at or shortly after a peak in pre-mRNA accumulation and increased mRNA production, suggesting bursts of splicing at these times.

**Figure 6:**
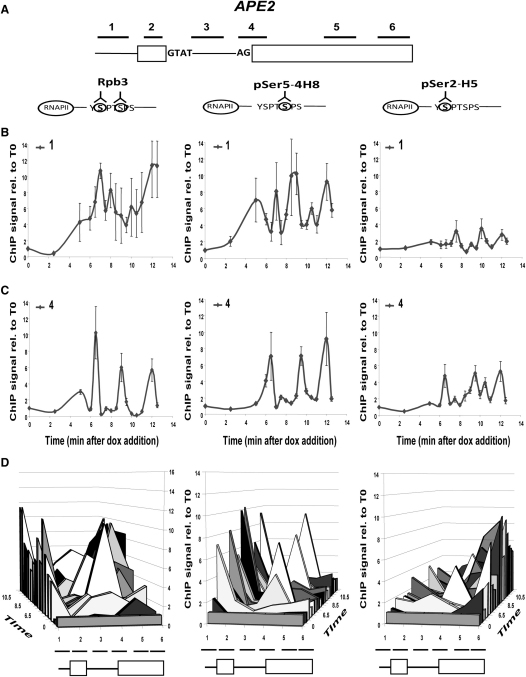
RNAPII Pauses Repeatedly at the 3′ End of the *APE2* Intron ChIP was performed to measure RNAPII recruitment to the *APE2* gene after doxycyclin induction as described in Figure 1, amplifying the six regions indicated in (A). Results are shown for ChIP of RNAPII at the promoter (amplicon 1), 3′SS (amplicon 4), or at all positions tested (B–D, respectively), using antibodies to total RNAPII (anti-Rpb3p; left), pSer5 (4H8 antibodies; middle) or pSer2 (H5 antibodies; right). Error bars indicate the standard error for qPCR performed in triplicate. For more detail, see also Figure S5.

The majority of intron-containing genes are constitutively expressed during normal growth in budding yeast. Therefore, in order to examine another intron-containing gene under similar induction conditions, the nonessential *APE2* gene was deleted from its genomic locus in the tetON yeast strain, and the *APE2* sequence, was integrated downstream of the doxycyclin-inducible promoter at the *HIS3* locus (like Ribo1). An advantage of *APE2* for this analysis is that both of its exons are longer than for Ribo1, allowing qPCR analysis of more, nonoverlapping, regions of the *APE2* gene. After the addition of doxycyclin, RNAPII occupancy on the gene was monitored as for Ribo1. As shown in Figure 6C, oscillations of RNAPII accumulation were observed in the region of the 3′SS, with a periodicity of 2.5 to 3 min. There was also increased RNAPII accumulation toward the 3′ end of the gene (Figure 6C, and more detail in Figure S5). Analysis of the phosphorylation status showed RNAPII with pSer5 in the promoter region and in the transient peaks over the 3′SS (Figure 6 and Figure S5). The pSer2 signal also increased from this point toward the 3′ end of the gene. Therefore, the *APE2* and Ribo1 genes show similar patterns of RNAPII accumulation after induction.

**Figure 7:**
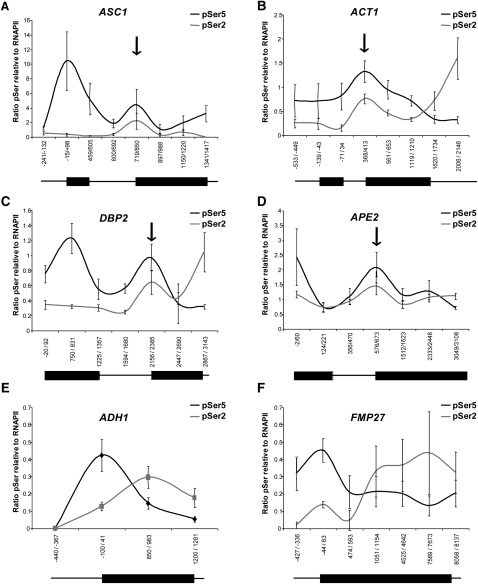
Phosphorylated RNAPII Accumulates around the 3′ Splice Sites of Endogenous Yeast Intron-Containing Genes ChIP analysis was performed to detect RNAPII with pSer5 (4H8 antibodies, black) or pSer2 (H5 antibodies, gray) along the lengths of the intron-containing genes *ASC1*, *ACT1*, *DBP2*, and *APE2* and the intronless genes *ADH1* and *FMP27*, all of which are constitutively expressed in yeast cells grown under steady-state conditions. Results are presented as the percentage of input relative to total RNAPII. Error bars indicate standard error for qPCR performed for three different cultures, each assayed in triplicate. Numbers indicate the positions of primers used for qPCR relative to the start codon of each open reading frame. Vertical arrows indicate the positions of the 3′SS, and in the line drawings, the thick lines indicate the positions of exons with respect to the PCR amplicons. For total RNAPII (anti-Rpb3p) ChIP data, see also Figure S6.

Although RNAPII pauses only very briefly at the end of the Ribo1 and *APE2* introns, the observation that pausing occurs repeatedly suggested that it may be possible to detect an elevated level of RNAPII over 3′ splice sites of constitutively expressed endogenous genes, without inducing synchronous transcription in the population of cells. However, with an asynchronous population of cells, the length, amplitude, and frequency of the pause or oscillation will determine how readily an elevated level of RNAPII will be detectable above background in the snapshot in time that is captured by the ChIP assay. Clearly, this may vary between genes. ChIP of RNAPII performed on four endogenous intron-containing genes, including *APE2*, shows that the level of RNAPII is slightly elevated over the 3′SS of all four genes, although the resolution is poor (Figure S6). As it had been shown that the level of phosphorylation of RNAPII is significantly elevated around the 3′SS of Ribo1 (Figure 2) and *APE2* (Figure 6), the amount of phosphorylated RNAPII was measured. Indeed, coincident peaks of enrichment of RNAPII with pSer5 and pSer2 are evident over the 3′ splice sites of all four intron-containing genes (Figures 7A–7D). It is particularly clear for *APE2* and *DBP2*, which have longer first exons, that the peak of pSer5 at the 3′SS is distinct from the peak at the promoter. In contrast, different patterns were observed for two intronless genes, *ADH1* and *FMP27*, with pSer5 declining and pSer2 increasing from the 5′ ends to the 3′ ends of the genes (Figures 7E and 7F).

## Discussion

The splicing-dependent transient accumulation of RNAPII was first detected in these experiments because we performed high-resolution time series analyses during the early stages of induction of an intron-containing gene. The detection of repeated pausing events lasting probably no more than 30 s each (Figures 5 and 6) indicates that RNAPII initiation and elongation on the doxycyclin-regulated Ribo1 and *APE2* genes are highly synchronous in different cells in the population, at least for the duration of these experiments. In principle, the accumulated RNAPII signal could represent sites of RNAPII arrest and premature transcription termination. However, the amount of Ribo1 mRNA increased very rapidly and more or less continuously, usually reaching a level as high or higher than for ILRibo1. Thus, there was no evidence of a defect in transcription and we propose that RNAPII pauses at a splicing-dependent transcriptional checkpoint.

The timing of the first observed pause after induction of the Ribo1 gene correlates with the first detectable cotranscriptional splicing event, as judged by the cotranscriptional recruitment of U2 and U5 snRNPs (Figure 3) and accumulation of spliced mRNA (Figure 1B shows RNA accumulation in the same culture). Furthermore, in a longer experiment (Figure 5), three large peaks of RNAPII also occurred at or immediately after peaks in pre-mRNA accumulation and rapid rises in mRNA production, which we propose may represent bursts of splicing activity. ChIP of RNAPII at the promoter may also suggest bursts of transcriptional activity (Figure 5C), although the periodicity differs from that of splicing and RNAPII pausing. Synchronous bursts of transcription have been observed after induction of mammalian genes although with longer intervals than observed here (Métivier et al., 2008; Heim et al., 2009).

The ChIP assays with Ribo1 indicate transient accumulation of RNAPII associated with amplicons 3 and 4 that span 180 bp around the 3′SS of Ribo1 (40 bp at the 3′ end of the intron and 140 bp at the 5′ end of exon2). Although amplicons 3 and 4 have overlapping sequences, they do provide some discrimination, as seen by the detection of U2 snRNP recruitment only with amplicon 3, and of U5 snRNP recruitment only with amplicon 4 (Figures 3C and 3D). Therefore, RNAPII may accumulate over a large area around the 3′SS and downstream. Our data suggest that the RNAPII pausing at the 3′ end of introns is unlikely to be sequence specific (discussed below); therefore, it may occur wherever RNAPII happens to be on the gene at the time when a checkpoint is triggered by the spliceosome, and this may vary slightly between cells in a population. In order to explore this issue further, a method with higher resolution than ChIP-PCR will be required. Although the available genome-wide data lack the resolution and sensitivity to show a transient accumulation of RNAPII over 3′ splice sites, RNAPII was found to accumulate over internal exons in humans as well as plants, *Drosophila* and nematodes (Brodsky et al., 2005; Schwartz et al., 2009; Chodavarapu et al., 2010), and especially over alternatively spliced exons in humans (Brodsky et al., 2005). As exons in higher eukaryotes tend to be short, these observations could be explained by the accumulation of RNAPII near the 3′ splice sites.

The *APE2* and Ribo1 genes showed similar patterns of RNAPII phosphorylation after induction, with pSer5 RNAPII at the promoter, oscillating levels of pSer5 RNAPII in the region of the 3′SS, and with pSer2 first detected at this point and persistently high toward the 3′ end of the genes, but not displaying the obvious periodicity seen for pSer5 at the 3′SS. Examination of the proportion of total RNAPII that is phosphorylated indicates that RNAPII at the 3′SS is hyperphosphorylated compared to RNAPII at the promoter (Figure S2). Also, particularly for inducible *APE2*, little or no pSer5 RNAPII was detected between the promoter and 3′SS (Figure S5), suggesting that RNAPII at the 3′SS is newly phosphorylated. The evidence of hyperphosphorylation of RNAPII over the 3′ splice sites of constitutively expressed genes in a population of cells growing under steady-state conditions suggests that this may be a common event in yeast.

The finding of strongly phosphorylated RNAPII paused near the 3′ ends of the Ribo1 and *APE2* introns is compatible with regulated transcription. RNAPII with pSer5 CTD is generally found in the 5′ region of genes at transcription initiation and is also associated with promoter proximal pausing, whereas pSer2 is associated with elongating RNAPII (Buratowski, 2009). We propose the existence of a transcriptional checkpoint, in which RNAPII pauses transiently near 3′ splice sites with an associated phosphorylation of Ser5 in the CTD. Conceivably, phosphorylation of Ser2 of the CTD at the same position on the gene may be associated with release from the proposed checkpoint.

pSer5 is not commonly found within the body of a gene, but it is not unknown. For example, hyperphosphorylation of RNAPII and inhibition of transcriptional elongation was observed after ultraviolet-induced DNA damage that causes changes to cotranscriptional alternative splicing in mammalian cells (Muñoz et al., 2009). Also, human Brm, the ATPase component of the chromatin remodeling complex SWI/SNF, affects the alternative splicing of a number of transcripts, apparently by inducing the accumulation of RNAPII with pSer5 on the variant exons of affected genes (Batsché et al., 2006). This was proposed to reflect a decreased elongation rate or pausing of RNAPII by Brm that would, in turn, favor inclusion of the variable exons in the mRNA. Thus, Brm was proposed to mediate crosstalk between transcription and RNA processing by directly or indirectly affecting the phosphorylation status of RNAPII (reviewed by Kornblihtt, 2006).

Although mutation of the 5′SS, BS, or 3′SS of the Ribo1 reporter intron abolished pausing at the 3′SS, pausing was restored by suppression in *trans* of the splicing defect of the BSRibo1 mutant transcript. Thus, it seems to be the splicing event rather than the sequence of the intron that actually triggers RNAPII pausing. The failure of 3′SSRibo1 mutant to cause this pause suggests that it may be triggered as a consequence of the assembly of spliceosomes that are competent to perform the second step of splicing (or more specifically by the recruitment of a second step splicing factor), and it could be the completion of the second step reaction that releases the pause. Interestingly, with the 3′SSRibo1 mutant, there was a pronounced accumulation of pSer5 modified RNAPII on the exon1/5′SS region of the gene (Figure 2D, left panel, amplicon 2), and a reduced amount of total RNAPII at the promoter (Figures 1C and 1E) compared with the splicing-competent Ribo1 gene. We propose that this may indicate the existence of an earlier checkpoint that is triggered by the splicing defect of 3′SSRibo1 transcripts, possibly resulting in reduced promoter activity.

As spliceosome assembly takes place cotranscriptionally it seems likely that splicing factors may mediate the RNAPII pause either by direct interaction or via chromatin-associated factors. Many splicing factors have been shown to interact with RNAPII complexes, including yeast Prp40p (Morris and Greenleaf 2000), and human SR proteins and U1 snRNP components (Das et al., 2007). There have been several reports of splicing factors affecting transcription (for reviews, see Fededa and Kornblihtt, 2008; Pandit et al., 2008). For example human U snRNPs were shown to strongly stimulate RNAPII elongation through interaction with the transcription elongation factor TAT-SF1 that, in turn, interacts with elongation factor P-TEFb (Fong and Zhou, 2001). Human SC35 also appears to stimulate transcription through interaction with P-TEFb (Lin et al., 2008). The P-TEFb complex contains the CDK9 kinase that binds to pSer5 and phosphorylates Ser2 of the CTD. Depletion of SC35 caused a transcription elongation defect and RNAPII was observed to accumulate on the body of certain genes, with a concomitant reduction in P-TEFb recruitment and of pSer2. In view of our observations with yeast, we suggest that SC35 depletion may have triggered a transcriptional checkpoint response either directly through lack of interaction of SC35 with P-TEFb, or indirectly by causing a splicing defect at the affected genes.

The human SKIP protein is another factor that was reported to activate transcription through interaction with P-TEFb, in this case enhancing Tat-regulated elongation at the HIV-1 promoter (Brès et al., 2005). SKIP (Prp45p in yeast) is both a coregulator of transcription (Brès et al., 2005; Brès et al., 2009) and an essential component of spliceosomes (Albers et al., 2003; Makarova et al., 2004). SKIP is therefore a candidate coupling or checkpoint factor that could mediate functional links between the two processes. Other candidates are the DExD/H-box RNA helicases, eight of which are involved in splicing in budding yeast. There is evidence that several of these proteins function as splicing fidelity factors, determining whether splicing should proceed to the next stage or the RNA should be discarded (Burgess and Guthrie, 1993; Mayas et al., 2006; Xu and Query, 2007; Query and Konarska, 2006).

A splicing-dependent transcriptional checkpoint might exist at the 3′ ends of introns simply to promote cotranscriptional splicing or, especially in metazoans, to enhance use of that particular 3′SS. Alternatively, multiple transcriptional checkpoints might exist that function as part of a surveillance mechanism in response to signals from fidelity factors at different stages during the splicing cycle, from spliceosome assembly to release of the spliced products. This would suggest a highly complex series of interactions, involving many factors. The system described here, using high-resolution kinetic assays in an organism that is amenable to genetics, provides a means to investigate the mechanism behind the proposed checkpoint(s) and identify the factors involved.

## Experimental Procedures

The Ribo reporter genes under control of a tet-O7/*CYC1-UAS* promoter (Bellí et al., 1998) were integrated at the *his3* locus in the tetON strain YIK91 or in the tetOFF strain YIK120 (Alexander et al., 2010). The doxycyclin-inducible *APE2* gene was constructed in a similar way in the tetON strain. See the Supplemental Information for details of strains (Table S1) and sequences of the Ribo reporter genes. Cultures were grown in synthetic dropout (SD) medium (Foremedium) at 30°C and doxycyclin was added to 4 μg/ml to induce or repress reporter gene expression. For RNA extraction, 10 ml aliquots of culture were pipetted into 5 ml of methanol at −70°C, pelleted, and stored at −70°C. RNA extraction (Tollervey and Mattaj 1987) and RT-qPCR were performed as described in the Supplementary Experimental Procedures, using primers as in Table S2. For ChIP analysis, 40 ml aliquots of culture were crosslinked for 10 min with 1% (v/v) formaldehyde and treated as described at http://www.ribosys.org/, using antibodies against Rpb3p (Neoclone), 4H8 antibodies (Millipore), or H5 antibodies (Covance). The DNA fragments (average size 350 bp) were amplified by qPCR using primers listed in Tables S2 and S3. ChIP experiments to detect phosphorylated RNAPII epitopes were performed at sub-saturating antibody titers (5-fold less than recommended by Kim et al. [2009]). ChIP data for the kinetic experiments are presented as percentage of input relative to uninduced level at T0. ChIP data for pSer RNAPII in Figure 7 and Figure S2 (right panels) are presented as the percentage of input relative to total RNAPII. Experiments presented in Figures 5 and 6 were performed in biological duplicate, and all other experiments were performed at least in triplicate, with all qPCR assays also performed in triplicate. In each case, a representative experiment is shown.

## Supplemental Information

Document S1. Supplemental Experimental Procedures, Six Figures, and Three Tables
