# Supplementary material for: Splicing-Dependent RNA Polymerase Pausing in Yeast
Source: Mol Cell. 2010 Nov 24;40(4):582–93. doi: 10.1016/j.molcel.2010.11.005 (PMC3000496; doi:10.1016/j.molcel.2010.11.005)
Supplement: Document S1. Supplemental Experimental Procedures, Six Figures, and Three Tables [file mmc1.pdf]

Molecular Cell, ***Volume 40***

Supplemental Information

Splicing-Dependent RNA Polymerase Pausing in Yeast

Ross D. Alexander, Steven A. Innocente, J. David Barrass, and Jean D. Beggs

Supplemental Experimental Procedures

Measurement of RNA by reverse transcription-quantitative PCR (RT-qPCR)

The RT and qPCR reactions are standardised using known amounts of in vitro transcribed RNAs with the same sequences, after mixing with total RNA extracted from uninduced cells. Prior to the conversion to cDNA, 10 µg of total RNA was treated with DNase1 (0.9U RQ1, Promega) according to the manufacturers protocol. cDNA was prepared from 5µg of the DNase treated RNA in a 10 µl reaction mixture containing 5x First strand synthesis buffer, 0.1M DTT, 10U RNase inhibitor (Roche), 10mM of each dNTP, 250nm Ribo1 specific primers (5_R and 6_R) and 7.5U Thermoscript RNase H (Invitrogen). After cDNA synthesis residual RNA was hydrolysed by the addition of 15 µl of 0.1mg/ml RNaseA and incubation at 37°C for 1 hour. cDNA was then diluted 1 / 20. qPCRs were performed in triplicate with SYBR green Jumpstart Taq ready mix (Sigma) in a Stratagene MX3005P real-time PCR machine. Reaction volumes were 10 µl, (5 µl 2x SYBR green qPCR mix and 300nm each primer and 1/1000th volume of Rox, 4 µl of cDNA template). Cycling parameters were 2min at 94°C, then 50 cycles of 10s at 94°C, 10s at 63°C and 20s at 72°C..

RiboSys reporter gene sequences and flanking sequences, showing the positions of oligonucleotides used as primers for RT and PCR.

tetO7-CYC1-UAS promoter sequence upstream of the reporter genes:

Arrows indicate the positions of oligos used as primers, pointing 5’ to 3’. Numbering is relative to the ATG, at the start of the open reading frame (shown in bold).
Colour code: tetO7; *CYC1*; *ACT1*

-558 TGACCACACC TCTACCGGCA GATCAATTCC TCGATCGAGT TTACCACTCC

-508 CTATCAGTGA TAGAGAAAAG TGAAAGTCGA GTTTACCACT CCCTATCAGT

-458 GATAGAGAAA AGTGAAAGTC GAGTTTACCA CTCCCTATCA GTGATAGAGA

-408 AAAGTGAAAG TCGAGTTTAC CACTCCTCAG TGACTATAGA GAAAAGTGAA

-358 AGTCGAGTTT ACCACTCCCT ATCAGTGATA GAGAAAAGTG AAAGTCGAGT

-308 TTACCACTCC CTATCAGTGA TAGAGAAAAG TGAAAGTCGA GTTTACCACT

1_F

-258 CCCTATCAGT GATAGAGAAA AGTGAAAGTC GAGCTCGGTA CCCTATGGCA

1_F

-208 TGCATGTGCT CTGTATGTAT ATAAAACTCT TGTTTTCTTC TTTTCTCTAA

-158 ATATTCTTTC CTTATACATT AGGTCCTTTG TAGCATAAAT TACTATACTT

2'_F

2_F

-108 CTATAGACAC GCAAACACAA ATACACACAC TAAATTACCG GATCAATTCG

2'_F

2_F

-58 GGGGATCGAC GGTATCGATA AGCTTGATAT CGAATTCCGA AAATTTACTG

1_R

-8 AATTAACA^{1}**ATG**

RiboSys reporter constructs are based on hybrid *ACT1-PGK1* sequences described previously (Hilleren and Parker 2003), with the addition of two λ boxB and six MS2 repeat sequences as indicate below.

Sequence of Ribo1:

-27 GAATTCCGAA AATTTACTGA ATTAACA^{1}**ATG** TCTTTATCTT CAAAGATCTG

24 GATTCTG***G***TA TGTTCTAGCG CTTGCACCAT CCCATTTAAC TGTAAGAAGA

2_R

6_R*

6_F

74 ATTGCACGGT CCCAATTGCTCGATGGGCCCTGAAGAAGGGCCCTAATCTC

6_F

124 GATGGGCCCTGAAGAAGGGCCCTAATCTCGAGAGATTT CTCTTTTACC TTT

174 TTTTACTATTTTTCACT CTCCCATAAC CTCCTATATT GACTGATCTG TAA

224 TAACCACGATATTATTG GAATAAATAG GGGCTTGAAA TTTGGAAAAA AAA

274 AAAAACTGAAATATTTT CGTGATAAGT GATAGTGATA TTCTTCTTTT ATT

3_F

324 TGCTACTGTT***ACTAAGT*** CTCATGTAC***T*** AACATCGATT GCTTCATTCTTTT

3_F

6_R*

4_F

374 TGTTGCTATATTATATGTTTA***G***ATCTCC CATGTCTCTA CTGGTGGTGG TG

4_F

2'_R

424 CTTCTTTGGAATTATTGG AAGGTAAGGA ATTGCCAGGT GTAGCTTTCT TA

2'_R 3_R

474 TCCGAAAAGAAAACTAGT TTAATTAACA TCTTTTACCC ATACGATGTT CC

4_R

524 TGACTATGCGGGCTATCC CTATGACGTC CCGGACTATG CAGGATCCTA TCC

4_R 5_F

574 ATATGACGTTCCAGATT ACGCTGCTCA GTGC**TGA** GGC GCGCCATTGA ATT

624 GAATTGAAATCGACCTA GCTAGCAGATCCTAAGGTACCTAATTGCCTAGA

5_R

674 AAACATGAGGATCACCCATGTCTGCAGGTCGACTCTAGAAAACATGAGGA

724 TCACCCATGTCTGCAGTATTCCCGGGTTCATTAGATCCTAAGGTACCTAA

774 TTGCCTAGAAAACATGAGGATCACCCATGTCTGCAGGTCGACTCTAGAAAA

824 CATGAGGATCACCCATGTCTGCAGTATTCCCGGGTTCATTAGATCCTAAG

874 GTACCTAATTGCCTAGAAAACATGAGGATCACCCATGTCTGCAGGTCGAC

924 TCCAGAAAACATGAGGATCACCCATGTCTGCAGTATTCCCGGGTTCATTA

974 GATCCCCTAGCTAGCATTCCCGAT AGATCAATTT TTTTCTTTTCTCTTTC

1024 CCCATCCTTTACGCTAAAATAATAG TTTATTTTAT TTTTTGAATA TTTTT

1074 TATTTATATACGTATATATAGACTA TTATTTATCT TTTAAGATTA TTAAG

1124 ATTTTTATTAAAAAA AAATTCGCCCC TTTTAATGCC TTTATGCAGT TTTT

1174 TTTCCCATTCGATATTTCTATGTCGG GTCAGCGTAT TTTAAGTTTA ATAA

1224 CTCGAA AATTCTGCGTTCGTTAGCGGCCGC

Explanation of reporter gene components:

ACT1 5' UTR (21 bases)

First coding exon = PGK1/ ACT1 fusion

ACT1 intron (**31** to **395**) containing two copies of λ boxB sequence (**93** to **150**)
Second exon = mini PGK1; 3 copies of HA (**500** to **605**)
6 copies of MS2
PGK1 3'UTR – positions of 3' end cleavage sites are underlined (Alexander et al., 2010)

Mutant variants (sequence changes shown bold and italicised above):
5'SSRibo1: 5' splice site (**31**) mutated to **C**.
BSRibo1: T at (**350**) mutated to **A** and cryptic branchsite **ACTAAGT** removed.
3'SSRibo1: 3' splice site (**395**) mutated to **C**.

Sequence of intronless reporter, ILRibo1:

2'_F →
AAATTACCG GATCAATTCGGGGGATCGAC GGTATCGATA AGCTTGATAT C
← 1_R

GAATTCCGAA AATTTACTGA ATTAACA[1]**ATG** TCTTTATCTT CAAAGAT CTCC

4_F →
CATGTCTCTA CTGGTGGTGG TGCTTCTTTGGAATTATTGG AAGGTAAGGA ATTGCCAGGT
← 2'_R

GTAGCTTTCT TATCCGAAAAGAAAACTAGT TTAATTAACA TCTTTTACCCATA

CGATGTT CCTGACTATGCGGGCTATCC CTATGACGTC CCGGACTATG CAGGATCCTA
← 4_R 5_F →

TCCATATGACGTTCCAGATT ACGCTGCTCA GTGC**TGA** GGC GCGCCATTGA

ATTGAATTGAAATCGACCTA GCTAGC AGATCCTAAGGTACCTAATTGCCTAGA
← 5_R

AAACATGAGGATCACCCATGTCTGCAGGTCGACTCTAGAAAACATGAGGA

TCACCCATGTCTGCAGTATTCCCGGGTTCATTAGATCCTAAGGTACCTAA

TTGCCTAGAAAACATGAGGATCACCCATGTCTGCAGGTCGACTCTAGAAAA

CATGAGGATCACCCATGTCTGCAGTATTCCCGGGTTCATTAGATCCTAAG

GTACCTAATTGCCTAGAAAACATGAGGATCACCCATGTCTGCAGGTCGAC

TCCAGAAAACATGAGGATCACCCATGTCTGCAGTATTCCCGGGTTCATTA

GATCCCCTAGCTAGCATTCCCGATAGATCAATTT TTTTCTTTTCTCTTTCCCCAT

CCTTTACGCTAAAATAATAGTTTATTTTATTTTTTGAATATTTTTTATTTATATAC

GTATATATAGACTATTAT TTAT CT TTTAATGATTATTAAGATTTTTATTAAAACA

AAATTCGCTCCTCTTTTAATGCCTTTATGCAGTTTTTTTTTCCCATTCGATATTTCTATGTCGG

GTCAGCGTAT TTTAAGTTTA ATAACTCGAA AATTCTGCGTTCGTTAGCGGCCGC

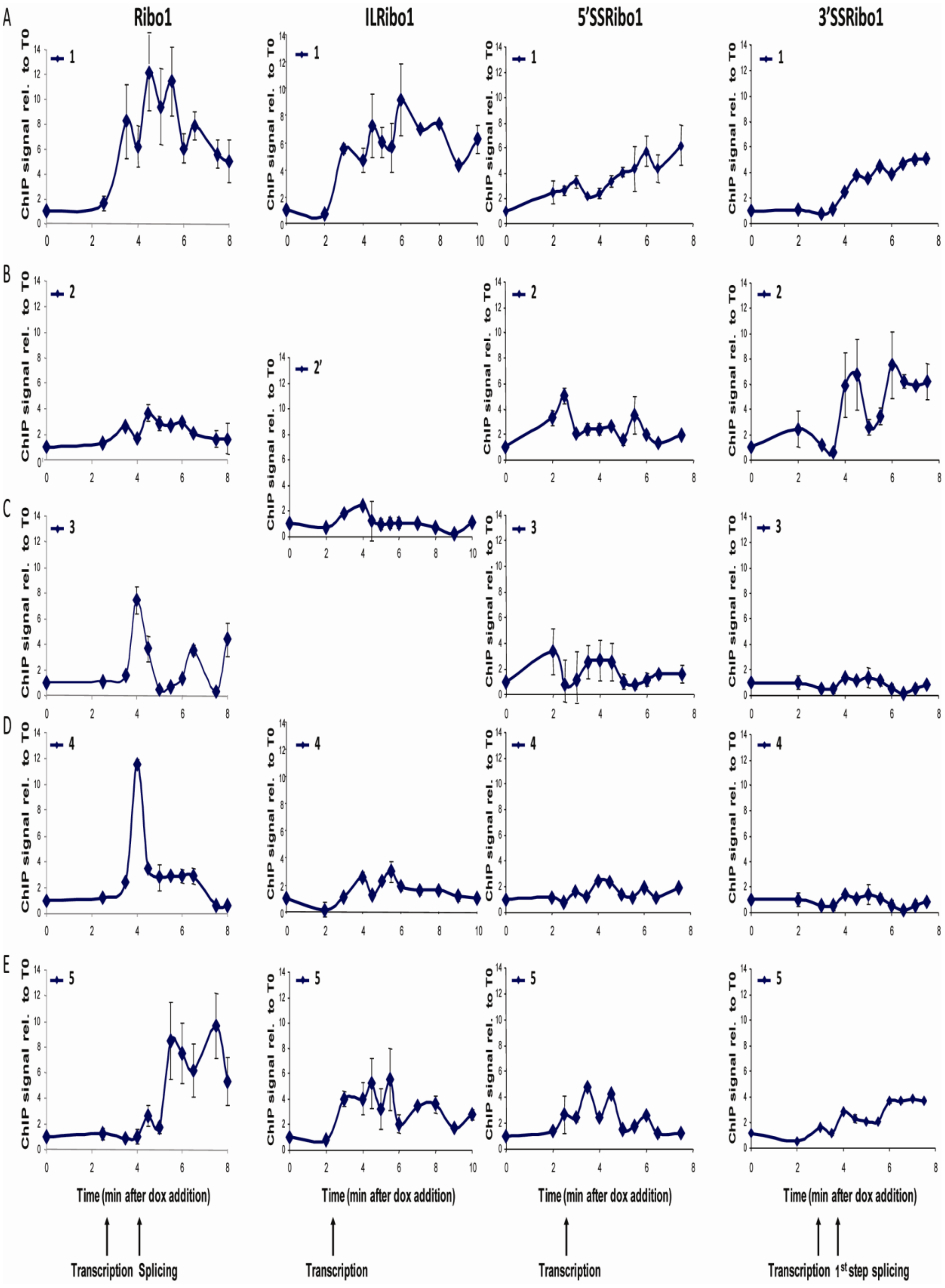

Figure S1, related to Figure 1:
ChIP of RNAPII (anti-Rpb3p) on Ribo1, ILRibo1, 5'SSRibo1 and 3'SSRibo1, at all positions along the genes and for all time points tested. Details are as described in Figure 1. Error bars indicate standard error for qPCR performed in triplicate

.

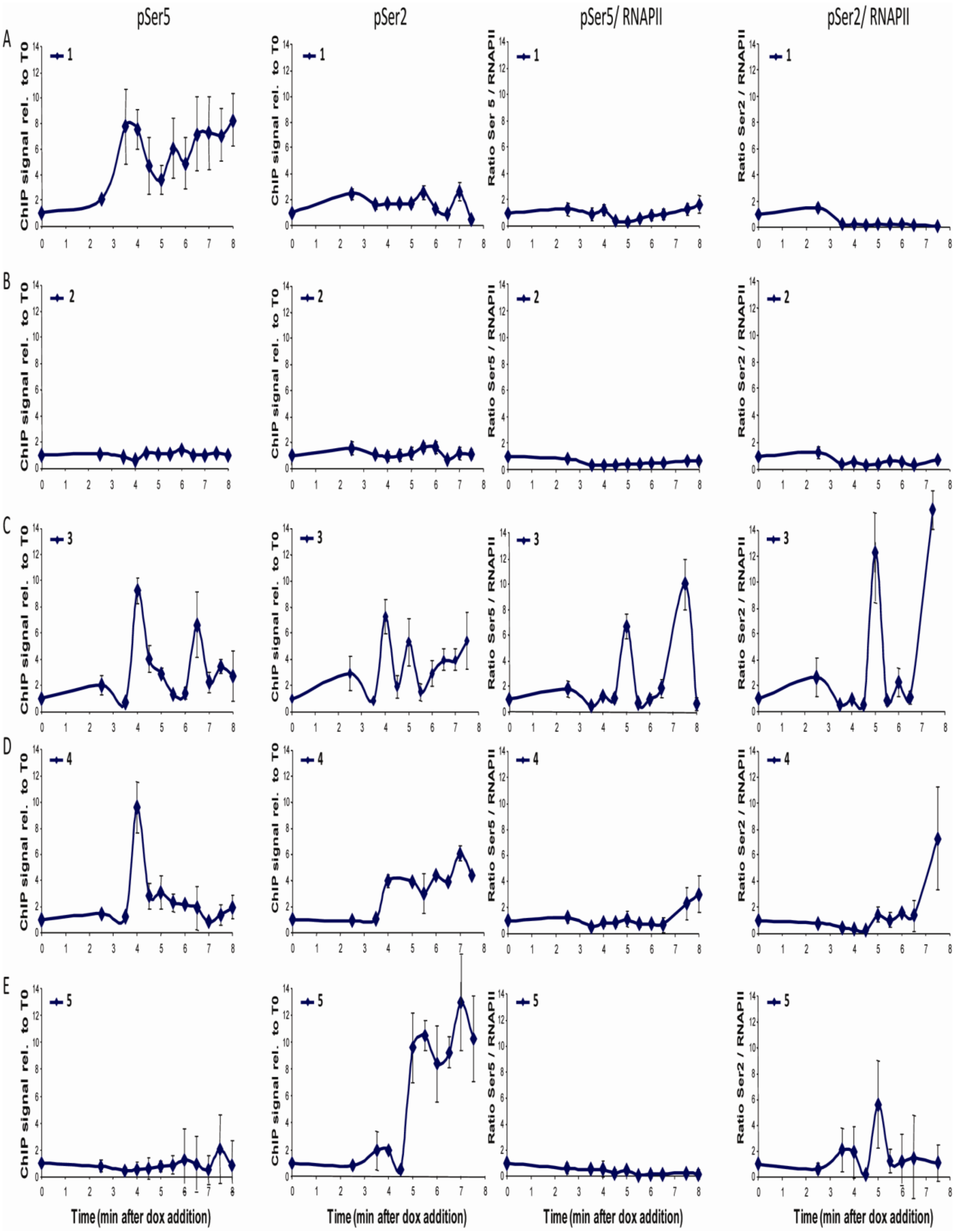

Figure S2, related to Figure 2:
ChIP of RNAPII that has PSer5 or PSer2 on the Ribo1 gene. ChIP data are shown for all positions along the gene and for all time points tested. The left pair of panels show data relative to T0. The right pair of panels show PSer relative to total RNAPII (anti-Rpb3p). Error bars indicate standard error for qPCR performed in triplicate

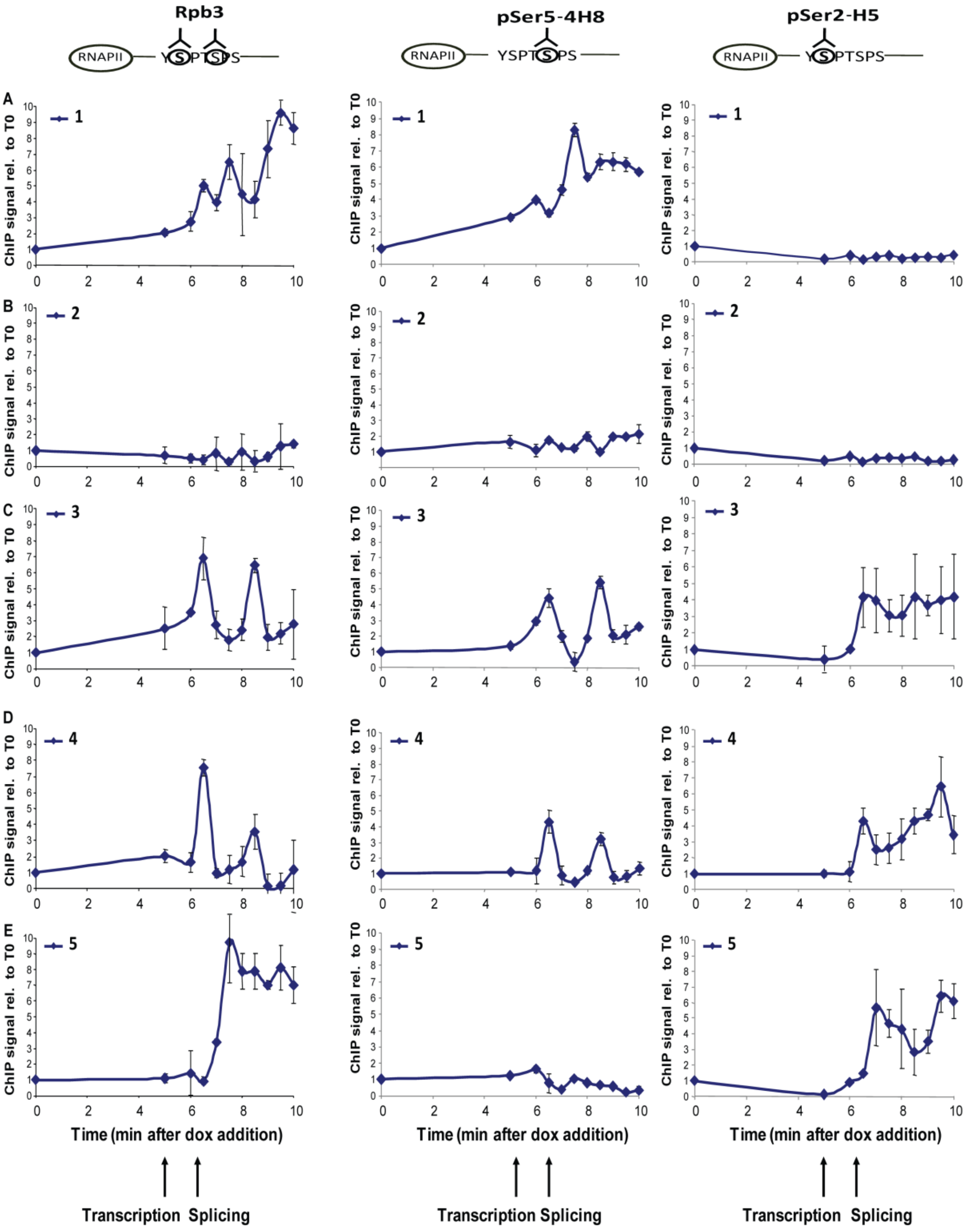

Figure S3, related to Figure 4:
ChIP of RNAPII during splicing with the BSRibo1 defect suppressed. The splicing defect in the BSRibo1 mutant reporter strain was suppressed by introduction of a plasmid encoding a U2 snRNA bearing a compensatory mutation that is complementary to the BS mutation. Left panel, total RNAPII (anti-Rpb3p); Middle panel, pSer5 (4H8 antibodies); Right panel, pSer2 (H5 antibodies), all relative to T0. Error bars indicate standard error for qPCR performed in triplicate

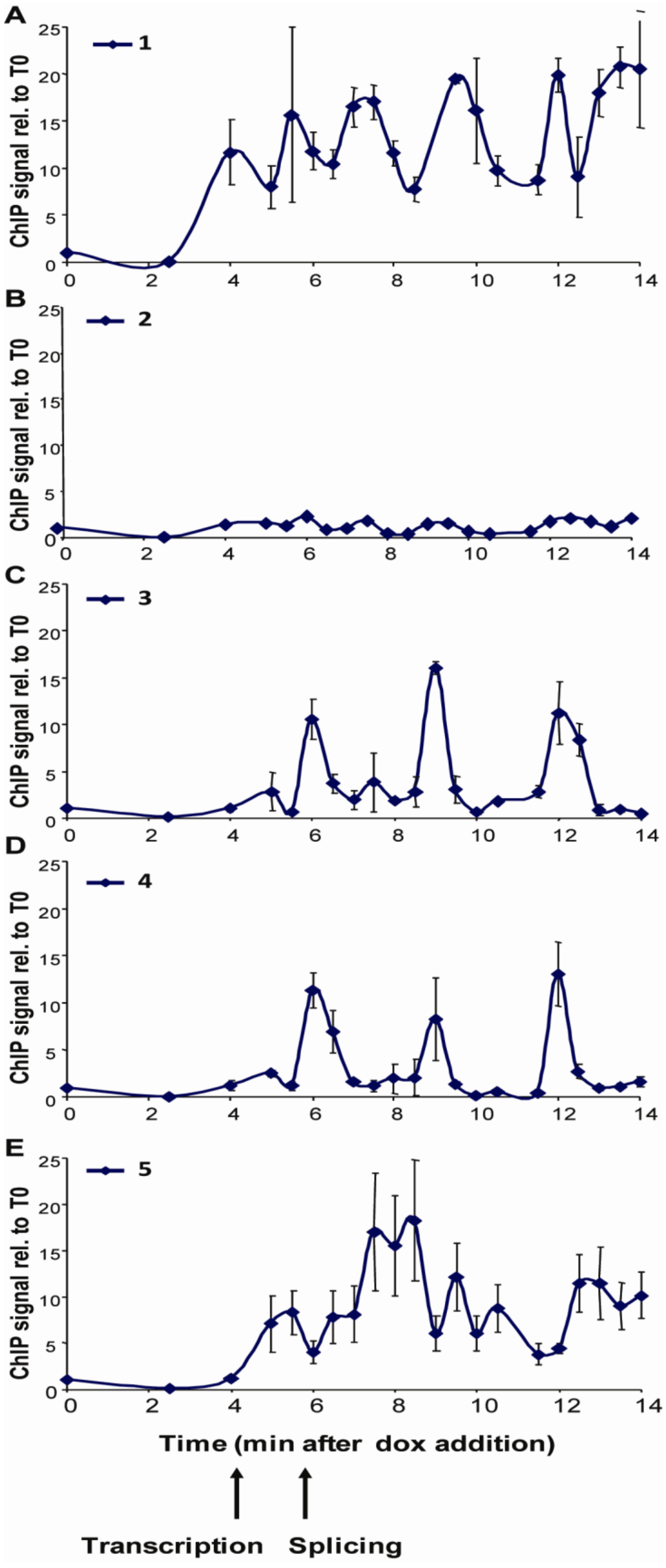

Figure S4, related to Figure 5:
ChIP of RNAPII (anti-Rpb3p) on Ribo1 at all positions and all time points tested during a longer period of induction. Error bars indicate standard error for qPCR performed in triplicate

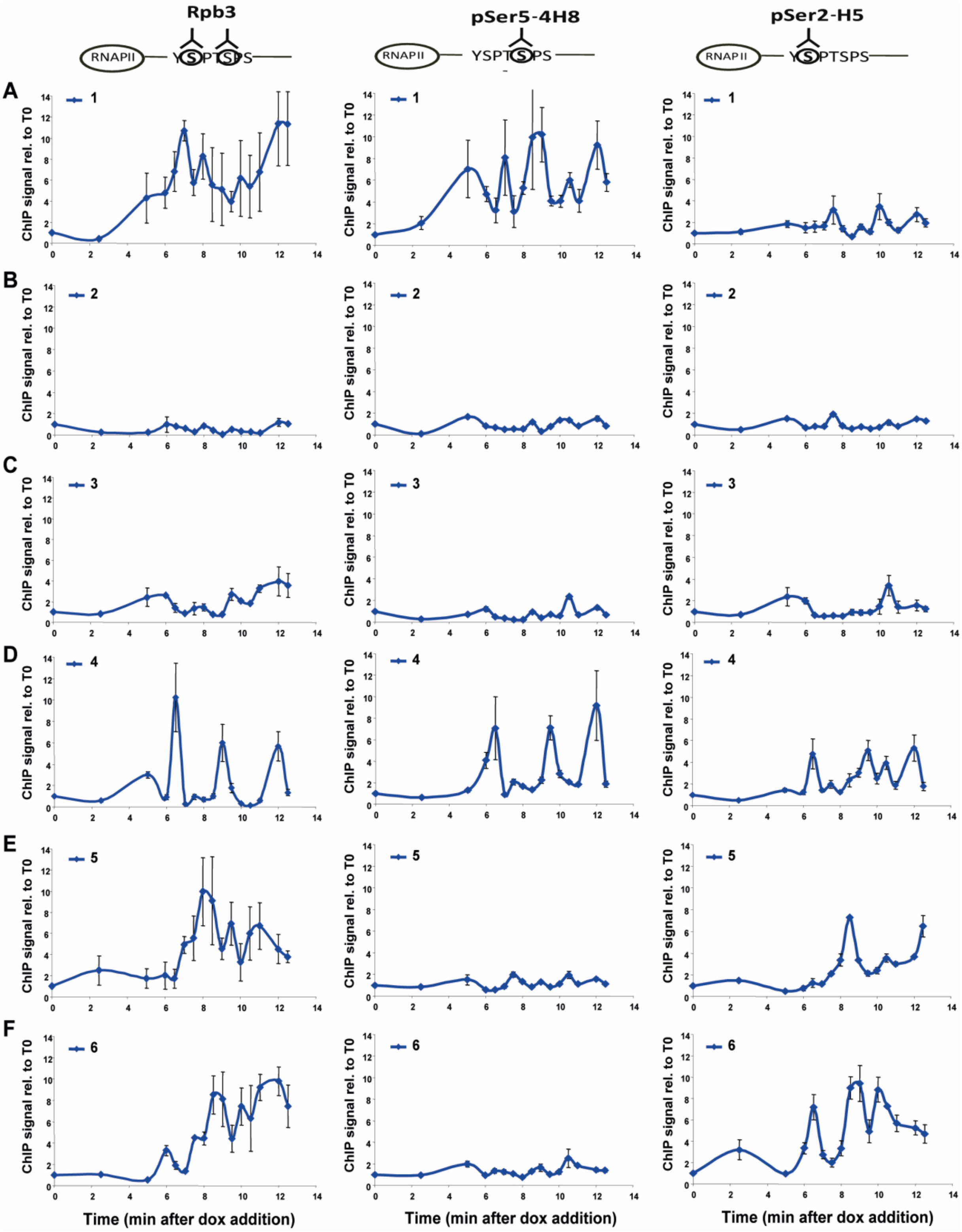

Figure S5, related to Figure 6:
ChIP of RNAPII with and without pSer5 or pSer2 on *APE2*
ChIP data are shown for all positions along the *APE2* gene and for all time points tested. Left column, total RNAPII (anti-Rpb3p); Middle column, pSer5 (4H8 antibodies); Right column, pSer2 (H5 antibodies), all relative to T0. Error bars indicate standard error for qPCR performed in triplicate

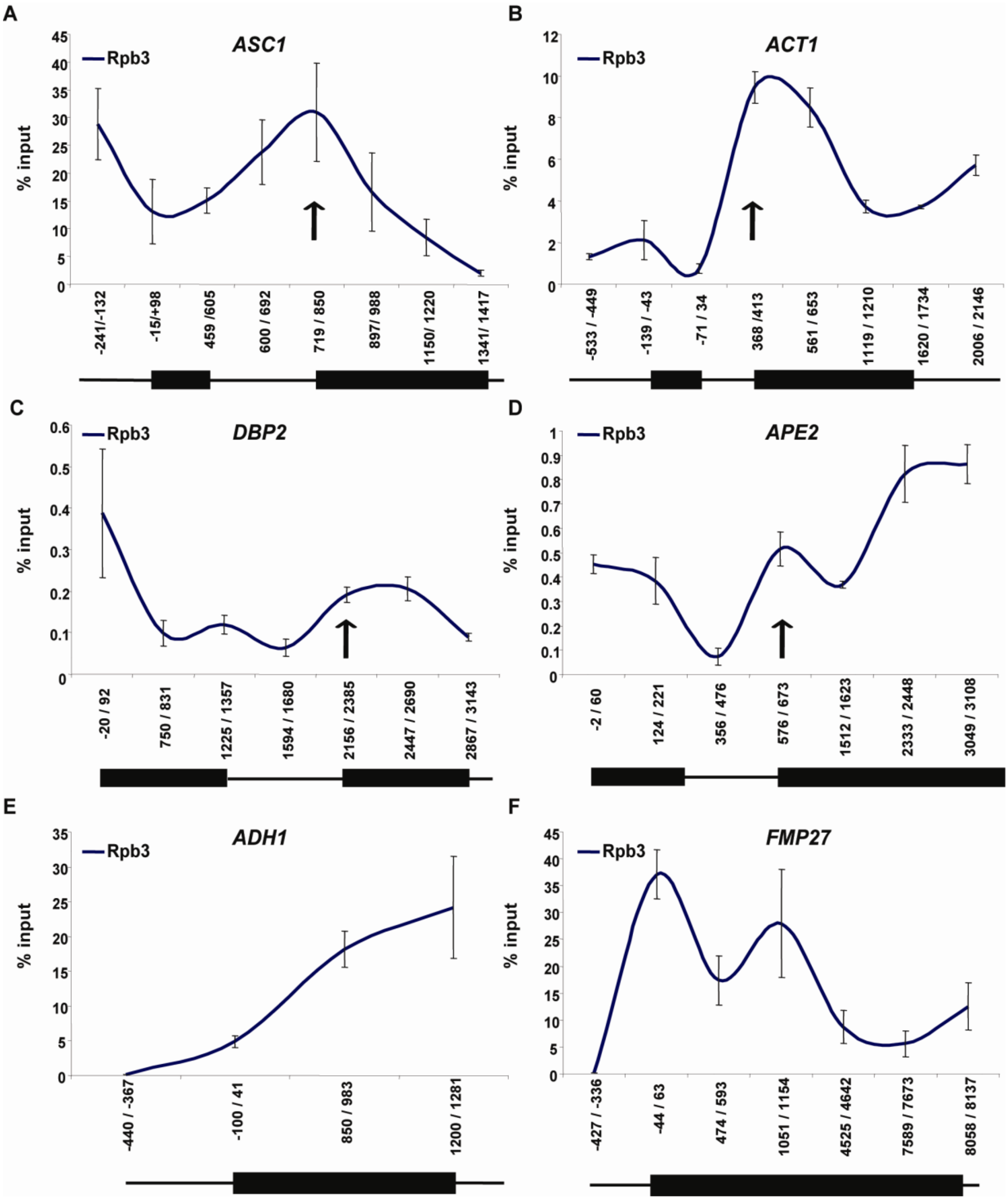

Figure S6, related to Figure 7:
ChIP of RNAPII (anti-Rpb3p) on four intron-containing and two intronless endogenous yeast genes. ChIP analysis was performed to detect RNAPII along the lengths of the intron-containing genes, *ASC1, ACT1, DBP2* and *APE2*, and the intronless genes *ADH1* and *FMP27*, all of which are constitutively expressed in yeast cells grown under steady state conditions. Results are presented as the percentage of input. Vertical arrows indicate the positions of the 3'SS, and in the line drawings, the thick lines indicate the positions of exons with respect to the PCR amplicons. Error bars indicate standard error for qPCR performed for three different cultures, each assayed in triplicate.

Table S1. Yeast Strains, Related to the Experimental Procedures

Strain	Genotype	Comment
W303	*MAT***a**, *his3-11,-15, leu2-3,-112, trp1-1, ura3-1, ade2-1, can1-100*	parent strain
YIK120	*Mat***a***, ade 2-1, trp1-1, leu2-3,-112, his3-11,-15, can1-100* *lys2:: tTA, ura3-1***::***tetR'-SSN6, Hph* W303 plus tTA in *LYS2*, *tetR'-SSN6, Hph* in *ura3*	tetOFF strain (Alexander et al., 2010)
YRA13	*his3::* tetO7-*CYC1-UAS-BSRibo1-Nat* otherwise as YIK120. p20-WT (*CEN, LEU2, SNR20*)	tetOFF BSRibo1* with p20-WT(Parker et al., 1987)
YRA14	*his3::* tetO7-*CYC1-UAS-BSRibo1-Nat* otherwise as YIK120. (*CEN, LEU2,* snr20-U36)	tetOFF BSRibo1* with p20-U36 (Parker et al., 1987)
YIK91	W303, *leu2::*P_{adh1}*-tetR-SSN6-LEU2*, p414(*CEN,TRP1*, P_{ADH1}*-tTA'*)	tetON strain derived from W303 (Alexander et al., 2010)
YIK91/Ribo1	*his3::* tetO7-*CYC1-UAS-Ribo1-Nat* otherwise as YIK91	tetON Ribo1*
YIK91/ILRibo1	*his3::* tetO7-*CYC1-UAS-ILRibo1-Nat* otherwise as YIK91	tetON ILRibo1*
YIK91/5'SSRibo1	*his3::* tetO7-*CYC1-UAS-5'SSRibo1-Nat* otherwise as YIK91	tetON 5'SSRIbo1*
YIK91/3'SSRibo1	*his3::* tetO7-*CYC1-UAS-3'SSRibo1-Nat* otherwise as YIK91	tetON 3'SSRibo1*
YIK91/APE2	*his3::* tetO7-*CYC1-UAS-APE2-Nat* , *ape2Δ0::*Kan^r otherwise as YIK91	tetON *APE2**

*The Ribo reporter genes with tetO7-*CYC1-UAS* and *HIS3* flanking sequences were integrated in the *his3* locus of YIK120 or YIK91, selecting for *Nat* (Alexander et al., 2010).

For doxycyclin-regulated *APE2*, the *APE2* ORF was deleted from the genome in YIK91, replacing it by Kan^r, and the *APE2* ORF was inserted between tetO7-*CYC1-UAS* and the *HIS3* 3' UTR in pMK121 (Alexander et al., 2010), which was cleaved with Pme1 and integrated in the *his3* locus, selecting for *Nat*.

Table S2. Oligonucleotides used as primers for RT, qPCR and ChIP experiments with RiboSys reporters, Related to the Experimental Procedures

Oligos	Sequence (5' to 3')	Location to ATG
1_F	GGTACCCTATGGCATGCATGTG	-222
1_R	CAAGCTTATCGATACCGTCGATC	-32
2_F	AATTCGGGGGATCGACGGTA	-64
2_R	GGTGCAAGCGCTAGAACATACCA	51
2'_F	TTCGGGGGATCGACGGTAT	-62
2'_R	CCAAAGAAGCACCACCACCA	433
3_F	CGATTGCTTCATTCTTTTTGTTGC	357
3_R	CCTGGCAATTCCTTACCTTCCA	461
4_F	TGGTGGTGGTGCTTCTTTGG	414
4_R	CCCGCATAGTCAGGAACATCG	536
5_F	ACGTTCCAGATTACGCTGCTCAGT	579
5_R‡	ACCTTAGGATCTCCTAGCTACGTCGATTT	658
6_F	AGAAGGGCCCTAATCTCGATGG	107
6_R*	GCAAGCGCTAGA*ACATAC***A**TAGTACA	51/337

‡5_R was also used for cDNA synthesis

*6_R is complementary to the branch site (underlined) and 5'SS (italics), with A instead of T opposite the branched A (Vogel et al., 1997). The positions of the primers are also indicated on the sequences of the reporter genes below.

Table S3. Oligonucleotides used as primers for ChIP experiments with endogenous genes, Related to the Experimental Procedures

Name	Oligo Sequence (5' to 3')
Numbering relative to ATG	
Asc1 -241 F	GTGCTTCTCCAGCGAAAGTC
Asc1 -132 R	AAAGGAATAGCCCAATGCCAAA
Asc1 -15 F	TAAATAAAGTGAAAAATGGCATCTAACGAA
Asc1 +98 R	AATAGGTTTGGTTGACCAGCAGAAGTAG
Asc1 +459 (5SS) F	AGTCAGAGTTGTTCCAAACGAAAAAGCTGATGATG
Asc1 +605 (5SS) R	AAATCCACTTTTCTTCTTCTACTCGATTGTCATCA
Asc1 +600 F	GATTTGTGTATGCCATTCAAATGATGT
Asc1 +692 R	TTGGTGATAATTGGTATGTCTCATTCG
Asc1 +719 (3SS) F	CTCTGCTCTTCTCTTTACTCGTTATGTCAAAATGG
Asc1 +850 (3SS) R	TGAAGTCAGCTTCAATTTGGAATTGGTTTAAGTT
Asc1 +897 F	ACTTTGATTGCTTCCGCTGGTAA
Asc1 +988 R	CATCTTGGGCAGACAAAGTG
Asc1 +1150 F	CTTGGTCTGCTGACGGTCAAAC
Asc1 +1220 R	ATAACTTGCCAAACTCTAATGACG
Asc1 +1341 F	TCGTCATAGATTTCGAAGTAATGAAAGAAA
Asc1 +1417 R	TTTCGCAGCAAACAGAAAGCA
DBP2 -20 F	TAAGGCAAATTTAGAGCAAATATGACTTACGGTGG
DBP2 +92 R	TTTCTATCAGAGTTTCTTCCACCGCGGA
DBP2 +750 F	GTTCGCGACAGATCGGACAGTGAGATT
DBP2 +831 R	GCTTTGGAATATCGTGTCCGGAAATAGTCAT
DBP2 +1225 F	GGTAGATCCCCAATTATGGTTGCTACTGATGTG
DBP2 +1357 R	AGCCATTAGGAAATAGCTGTAATATCGTTAGGGGT
DBP2 +1594 F	AAAATTGACTTTAATTAGTCGTTTTGAGAGACGGG
DBP2 +1680 R	GACATGAAGTGCAAATCATGAACACAAATACACTT
DBP2 +2156 F	AACATGAATTTGTGGGGGGCATGAAAATA
DBP2 +2385 R	CCTGGCATATCGTAGTTGATAACGTAATTGATACC
DBP2 +2447 F	TGAAATACGACAGGAGATCTTATGGTGGCG
DBP2 +2690 R	TCTCTGCCTGTTACCGCCGTAACCA
DBP2 +2867 F	TAGGACAGACACTTTTCTTTGTTCTCGTACAACCC
DBP2 +3143 R	CCATAAGCGCTAGTGCACGACTTCTTTGTAAGT
Act1 -533 F	CCACAGCAATTAATGCACAACATTTA
Act1 -449 R	GGCATATGTTTTTAAGGGTTTTGAGG
Act1 -139 F	TTCCCCTTTCTACTCAAACCAAGAAG
Act1 -43 R	AAGCGTGAAAAATCTAAAAGCTGATG
Act1 -71 (5SS) F	TACATCAGCTTTTAGATTTTTCACGCTTACTGCTT
Act1 +34 (5SS) R	GATGGTGCAAGCGCTAGAACATACCAGAAT
Act1 +368 (3SS) F	TGTACTAACATCGATTGCTTCATTCTTTTTGTTGC
Act1 +413 (3SS) R	GACGATAGATGGGAAGACAGCACGAGGA
Act1 +561 F	ATCTGGCATCATACCTTCTACAACGA
Act1 +653 R	GTTTGATTTAGGGTTCATTGGAGCTT
Act1 +1119 F	TCTGCCGGTATTGACCAAACTACTTA

Act1 +1210 R	CCGGACATAACGATGTTACCGTATAA
Act1 +1595 F	ATGTGTTTTGTCTCTCCCTTTTCTACGAAAATTTC
Act1 +1620 F	CGAAAATTTCAAAAATTGACCAAAAA
Act1 +1727 R	ATGATACACGGTCCAATGGATAAACA
Act1 +1734 R	TGATCATATGATACACGGTCCAATGGATAAACAT
Act1 +2006 F	AAATCCCTTAACTTTCACTCGTGAGG
Act1 +2146 R	ATATTTGCCATGAGCCTTTCCAGTAT
APE2 - 2F	TATGCCAATTGTTCGGTGGCTA
APE2 + 60R	CCTAGGGTGTGCAGCAATAGACC
APE2 +124F	CCACCAGCAGGCGTCAGTAGAT
APE2 +221R	GGGCGTCTATTCATGATCTTCACA
APE2 +356F	AACATGAGACCGAAATACCAGGATG
APE2 + 470R	CCTGCTGCAGAAGATAAGAATATGAGG
APE2 +576F	TCTGCTCGTTACCGACCTTTGA
APE2 +673R	TTCACGATTTGGGGTTTTACTGG
APE2 +1512F	CCACCAGCAGGCGTCAGTAGAT
APE2 +1623R	GGGCGTCTATTCATGATCTTCACA
APE2 +2333F	CGGTCTGGTTGCTGATGTCAAG
APE2 +2448R	TTTGGTCCCAGACGACAAATGA
APE2 +3049F	TTTCAATGCTAGGCTCCGTCGT
APE2 +3108R	CCTTTCGTGGATTTAGTGGCAAA
FMP27 -427 F	TTCAATTGATCAAATTTATGGAAGATCCTCAAGAA
FMP27 -336 R	GGAACAAAAACTTGACAAATTTGAAACTCTGGAT
FMP27 -44 F	GAACATAAGAATCCTTAGAAAAGCCCTTTACCTCG
FMP27 +63 R	CCATAAGAAAGTCACTGCAAATATAAGCCACTTGT
FMP27 +474 F	AAGATTTGATTCCTTTTTGAGAAAACTTCTTTGGA
FMP27 +593 R	CCATCCTTCAGAGGATTCATAATTTCACCAATT
FMP27 +1051 F	TTGAATCTAAATCGAAAACATCAAAGCCACG
FMP27 +1154 R	AATTTTTGAGAGAACAAATTGGTTTCGCCA
FMP27 +4525 F	AGACCTAGTACCAATACAATGTTCATTCCAAACCA
FMP27 +4642 R	CCTTGTCTGCTTTTTCGTTTTTACTTGATGTAGTG
FMP27 +7589 F	TGAACAGCTTCAAACTTTGTATCAGTTATAAGGGC
FMP27 +7673 R	GGGAAATTGAAAACAAAGTTAGTAACGTTAGCCAA
FMP27 +8058 F	AAAGTAAAAAAAATAATGGTCTCTAGCGGGATCG
FMP27 +8137 R	CCCAGTTGGTTAAGGCACCGTGCTAATAAC
ADH1 -440 F	TTGCCATCTATTGAAGTAATAATAGGCGCATGCAA
ADH1 -362 R	AGACAACAACGGGGGAGAGAGAAAAGAAA
ADH1 -100 F	TTTGTTTCCTCGTCATTGTTCTCGTTCCC
ADH1 +41 R	TCGTAGAAGATAACACCTTTTTGAGTTTCTGGGAT
ADH1 +850 F	CAAGTCGTCAAGTCCATCTCTATTGTTGGTTCT
ADH1 +938 R	AAACCTCTGGCGAAGAAGTCCAAAGCTT
ADH1 +1200 F	GCATGAGGTCGCTCTTATTGACCACACCTCTACCGGCATG
ADH1 +1281 R	CAATTGGGTGAAATGGGGAGCGATT
